# Enhancing Transthyretin Binding Affinity Prediction with a Consensus Model: Insights from the Tox24 Challenge

**DOI:** 10.1021/acs.chemrestox.4c00560

**Published:** 2025-04-26

**Authors:** Xiaolin Pan, Yaowen Gu, Weijun Zhou, Yingkai Zhang

**Affiliations:** † Department of Chemistry, New York University, New York, New York 10003, United States; ‡ Simons Center for Computational Physical Chemistry at New York University, New York, New York 10003, United States; § NYU-ECNU Center for Computational Chemistry at NYU Shanghai, Shanghai 200062, China

## Abstract

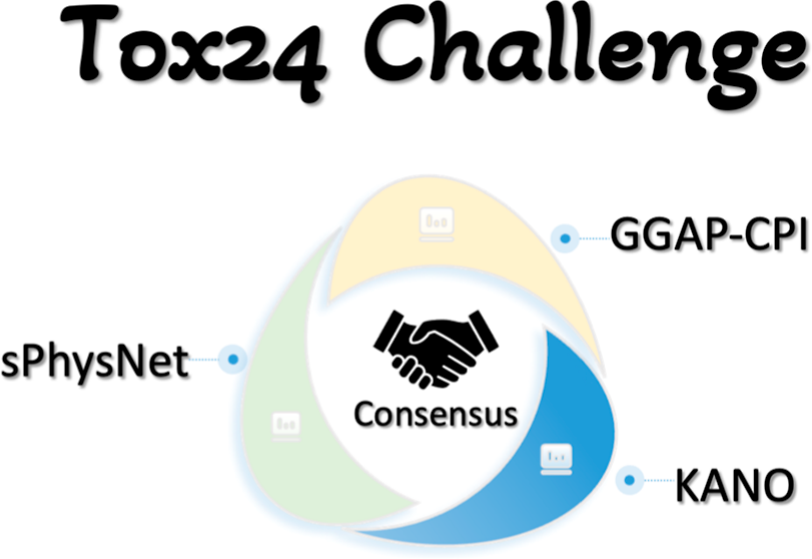

Transthyretin (TTR) plays a vital role in thyroid hormone transport and homeostasis in both the blood and target tissues. Interactions between exogenous compounds and TTR can disrupt the function of the endocrine system, potentially causing toxicity. In the Tox24 challenge, we leveraged the data set provided by the organizers to develop a deep learning-based consensus model, integrating sPhysNet, KANO, and GGAP-CPI for predicting TTR binding affinity. Each model utilized distinct levels of molecular information, including 2D topology, 3D geometry, and protein–ligand interactions. Our consensus model achieved favorable performance on the blind test set, yielding an RMSE of 20.8 and ranking fifth among all submissions. Following the release of the blind test set, we incorporated the leaderboard test set into our training data, further reducing the RMSE to 20.6 in an offlineretrospective study. These results demonstrate that combining three regression models across different modalities significantly enhances the predictive accuracy. Furthermore, we employ the standard deviation of the consensus model’s ensemble outputs as an uncertainty estimate. Our analysis reveals that both the RMSE and interval error of predictions increase with rising uncertainty, indicating that the uncertainty can serve as a useful measure of prediction confidence. We believe that this consensus model can be a valuable resource for identifying potential TTR binders and predicting their binding affinity in silico. The source code for data preparation, model training, and prediction can be accessed at https://github.com/xiaolinpan/tox24_challenge_submission_yingkai_lab.

## Introduction

Transthyretin (TTR), synthesized primarily in the liver and choroid plexus, is distributed throughout the bloodstream and cerebrospinal fluid. This protein is essential for maintaining the homeostasis of thyroid hormones in blood and tissues.^1^ The binding of exogenous compounds to TTR can competitively inhibit the transport of thyroid hormones (TH) to target tissues, disrupting TH-dependent biological pathways. Moreover, TH displaced from TTR can be metabolized and excreted, leading to a decrease in serum TH levels.^2^ Several in vitro assays can be used to evaluate competitive binding to TTR, including the fluorescence-based competitive binding assays using fluorescein isothiocyanate-T4 conjugate (FITC-T4) or 8-anilino-1-naphthalenesulfonic acid ammonium salt (ANSA)^3−5^ and a radiolabeled ligand displacement assay.^6^ However, these assays are typically high in cost and time consumption, limiting their capacity for large-scale, rapid screening.

Therefore, the development of accurate and rapid computational models for TTR binding is important for avoiding the toxicity of candidates in medicinal chemistry, food chemistry, and environmental chemistry applications. For instance, Kovarich et al.^7^ utilized a k-nearest neighbor (KNN)-based method to calculate the binding potency of compounds in competitive binding to TTR, while Rybacka et al.^8^ developed a classification model based on molecular descriptors calculated via OCHEM^9^ to predict compound–TTR interactions. Yang et al. combined in vitro experiments and computational methods to study the strength of binding and mechanisms of phenolic disinfection byproducts with TTR, employing multiple linear regression for QSAR modeling.^10^ Yang et al. also developed a combination method that contains both a regression and classification model.^11^ The classification model is employed to screen for molecules that disrupt human TTR, while the regression model is employed to calculate the binding activity. Additionally, Zhang et al. developed a structure-based virtual screening method that successfully identified potential thyroid-disrupting chemicals and determined the crystal complex structures of human TTR binding with several exogenous compounds.^12^

**Figure 1:**
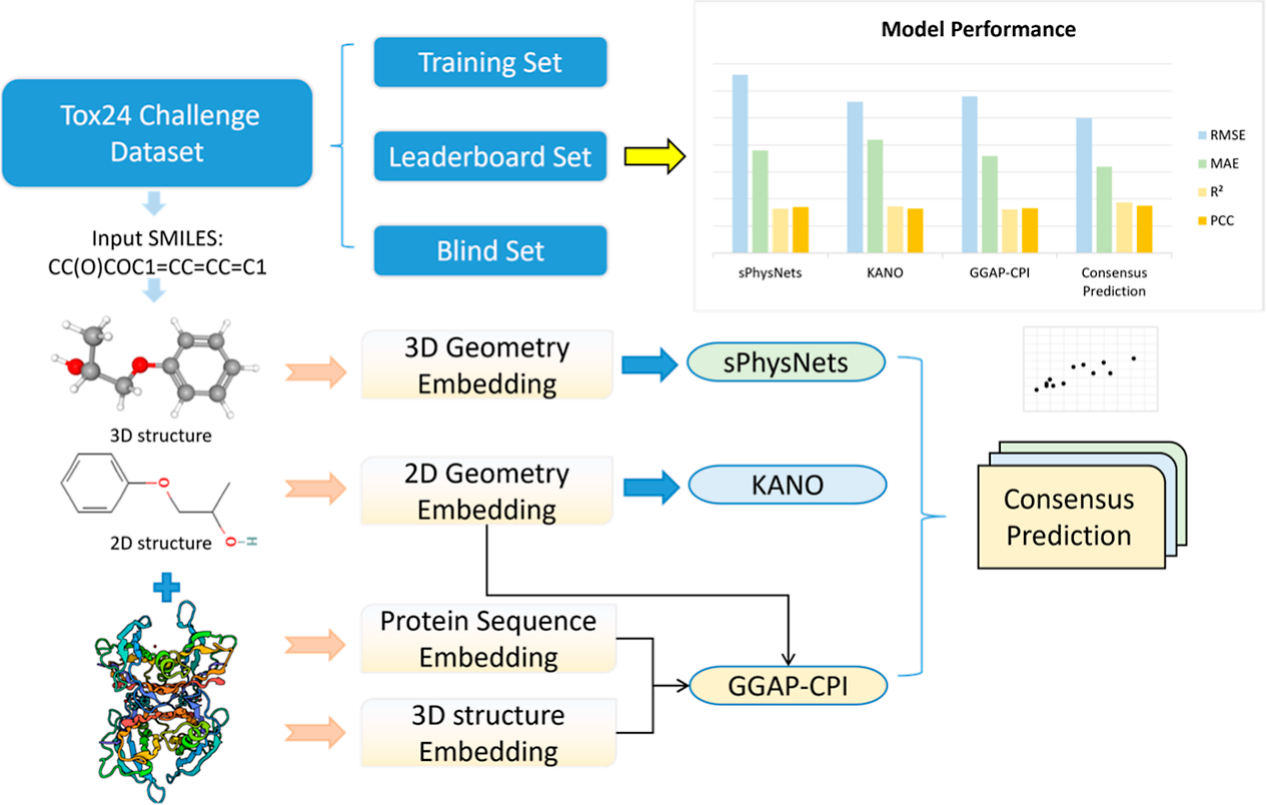
Schematic workflow of our approach for predicting TTR binding affinity in the Tox24 challenge. Final predictions are obtained from a consensus model integrating three distinct regression models: sPhysNet, KANO, and GGAP-CPI, each leveraging a different level of molecular information, including 2D topology, 3D geometry, and protein–ligand interactions.

In recent years, advances in artificial intelligence (AI) have spurred the development of numerous novel models for molecular property prediction.^13−22^ However, the accuracy of data-driven deep learning approaches heavily depends on both the quantity and quality of the available data set. The biggest data set in the previous study only contains 407 binding compounds of TTR,^11^ which poses a considerable challenge to develop high-confidence models in the community. In contrast, the extensive experimental data set provided by the Tox24 challenge offers a robust foundation for developing more accurate predictive models.^23,24^ In this challenge, our team developed a novel deep consensus model designed to improve the predictive accuracy of TTR binding activity by combining three distinct regression methods: simplified PhysNet (sPhysNet),^25,26^ knowledge graph-enhanced molecular contrastive learning with functional prompt (KANO),^27^ and protein graph and ligand graph network with attention pooling for compound–protein interaction prediction (GGAP-CPI)^28^ that incorporate various molecular levels of information, including 2D topology, 3D geometry, and protein–ligand interactions. Figure 1 shows the workflow of our consensus model building in detail. Our model demonstrated competitive performance on the blind test set, achieving a root-mean-square error (RMSE) of 20.8, which compares favorably with most of the other submissions and ranks fifth among all submissions.^29^ After incorporating the leaderboard set into our training data, we further improved the RMSE to 20.6 in an offline retrospective study. We believe that this model can serve as a valuable resource for identifying potential TTR binders and predicting their binding affinity in silico.

## Materials and Methods

### Data Preparation

The Tox24 challenge provides the largest data set of TTR binding affinity for model training and validation until now, including the training set, leaderboard test set, and blind test set. It contains 1012 compounds, 200 compounds, and 300 compounds, respectively. These compounds were screened from the U.S. EPA’s ToxCast libraries using human TTR protein and the fluorescent probe ANSA.^24^ This extensive experimental data set offers a robust foundation for developing more accurate predictive models. In this data set, some compounds are mixtures, salts, or contain metal elements. To ensure a cleaner data set, we standardized all compounds using RDKit, removing salts and generating canonical SMILES. For the record, with more than one molecule, we do not use it as training data. Finally, the training set contains 1002 compounds. For each compound, we generated 100 conformations using ETKDG^30,31^ in RDKit and selected the lowest energy conformation post-MMFF94 optimization.^32−36^ If ETKDG failed, we used Schrodinger as an alternative.

### Deep Learning Models

In this challenge, we developed three distinct models to estimate the TTR binding affinity of compounds, including sPhysNet,^25,26^ KANO,^27^ and GGAP-CPI.^28^ sPhysNet leverages 3D molecular geometry for property prediction by using radial basis functions (RBFs) to embed molecular features. KANO utilizes a chemical element-oriented knowledge graph (ElementKG) that integrates fundamental information on functional groups and element types to encode molecular information. GGAP-CPI combines both 2D ligand molecular graphs and 3D protein structure graphs with predrilled ligand and protein sequence embeddings. Each model underwent K-fold cross-validations (CVs) on the provided training set and was then tested independently on the leaderboard test set. Specifically, we employed a 5-fold split for sPhysNet and KANO and a 10-fold split for GGAP-CPI. During the blind challenge, the leaderboard test set was excluded from model training; however, in a subsequent retrospective study, it was merged with the training data. We evaluated our model performance using four commonly employed metrics: the coefficient of determination (*R*^2^), root-mean-square error (RMSE), mean absolute error (MAE), and Pearson correlation coefficient (PCC). These metrics were calculated by the following functions
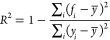

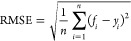

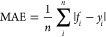

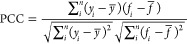


Here, *f*_*i*_ denotes the predicted value for molecule *i*, *y*_*i*_ represents its corresponding experimental value, *f̅* is the mean of all predictions, *y̅* is the mean of all experimental values, and *n* is the total number of molecules in the data set.

We then integrated these models into a consensus to enhance predictive accuracy on the blind test set, taking advantage of their diverse molecular embeddings and complementary strengths. The details of these three model architectures are described as follows.

### sPhysNet

This multitask deep learning model directly estimates electronic energies and solvation energies. In this model architecture, each atom in a compound is processed through an embedding matrix to generate node embeddings, while radial basis functions (RBFs) encode interatomic distances as edge embeddings. These embeddings are iteratively refined through three interaction modules that incorporate message passing, residual connections, and gate mechanisms. The final node embeddings are then used by the output layer to produce the desired properties. In this study, we fine-tuned a pretrained sPhysNet model with our training set. The learning rate was set to 0.005, and training was conducted for 800 epochs using the EmaAmsGrad optimizer, which was configured with beta parameters ranging from 0.0 to 0.999, without applying any weight decay.

### KANO

It is a recently developed deep learning framework that is designed for molecular property prediction. At its core, ElementKG integrates fundamental information about functional groups, element types, and their interrelationships. RDKit is used to identify functional groups by their names in ElementKG. A learnable vector is incorporated to capture the relative importance of these groups. Two self-attention layers generate embeddings for both the identified functional groups and the mediator vector. The outputs pass through an MLP to produce a functional prompt. This functional prompt is then combined with the molecular representation of each node, resulting in a prompt-enhanced molecular graph. That graph is fed into the pretrained graph encoder, leveraging the functional group knowledge gained during contrastive pretraining. Finally, a neural network is applied to the output for a molecular property prediction task. In this work, the pretrained KANO model was fine-tuned using our designated training set. The model was trained for 100 epochs by using a batch size of 256. It employed an initial learning rate of 5 × 10^–4^, a maximum learning rate of 1 × 10^–3^, and a final learning rate of 1 × 10^–4^, with the Adam optimizer managing the training process.

### GGAP-CPI

It is a protein Graph and ligand Graph with Attention Pooling for Compound–Protein Interaction prediction (GGAP-CPI) model that utilizes both 2D ligand molecular graphs and 3D protein structure graphs. It employs KANO as the ligand encoder and a graph convolutional network with pretrained ESM-2^37^ protein embeddings as the protein encoder. A cross-attention pooling module is used to simulate the protein–ligand interaction and integrate the learned ligand and protein representations, which are then processed by a multilayer perceptron (MLP) for toxicity prediction. GGAP-CPI is an interaction-based method in our approach since it learns protein and ligand representations based on protein residue–residue interactions (by Graphein^38^) and ligand atom–atom interactions from complex structure-free CPI data. To introduce protein structural variance, both the TTR crystal structure (PDB: 3D7P) and AlphaFold2-predicted^39^ structures were used. GGAP-CPI was optimized using the mean squared error as the loss function. Additional 215 TTR bioactivity end points are collected from public databases (EquiVS^40^ and Papyrus^41^) to benefit GGAP-CPI training from a data perspective. These assessed ligands do not overlap with those in the Tox24 challenge test set. We applied min–max scaling to rescale the Tox24 data from approximately −50 to 120 into a range of 0–12, aligning it with the negative log(nM) bioactivity distribution. The Tox24 challenge training set and the collected data were then combined for GGAP-CPI training. The model is trained using the Adam optimizer with a learning rate of 1 × 10^–3^. The training process runs for 100 epochs with a batch size of 32. The model employs a hidden dimension of 300 nm and a dropout rate of 0.05 to prevent overfitting.

### Offline Testing

To fully leverage the available training data from the Tox24 challenge and enhance the robustness of our base models, we conducted an additional offline experiment using 5 × 5-fold cross-validation. In this setup, we combined the training and leaderboard sets for model training while ensuring that data splitting was consistent across sPhysNet, KANO, and GGAP-CPI. This strategy resulted in a total of 75 base models (25 per method), whose predictions were integrated via a soft voting ensemble (calculation of average values of all base model predictions) for the final prediction and inference.

## Results and Discussion

### Molecular Properties and Chemical Structure Distribution

**Figure 2:**
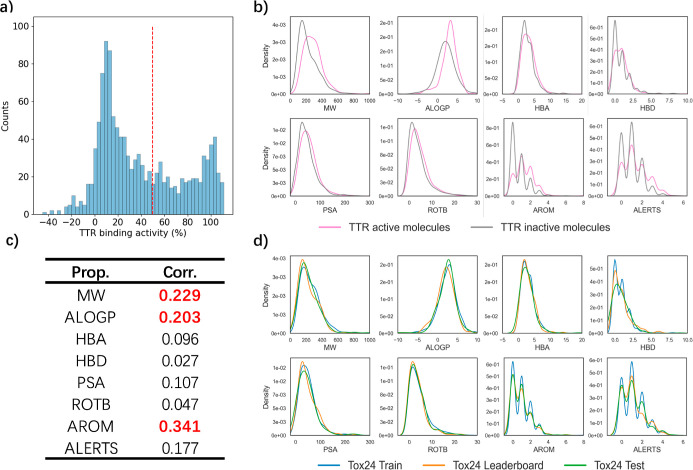
TTR binding activity and QED property distributions and correlations in Tox24 train + leaderboard data sets. (a) TTR binding activity (%) distribution; (b) QED (MW (Da): molecular weights; ALOGP: partition coefficient logP based on the Ghose–Crippen atomic method;^42^ HBA: number of hydrogen bond acceptors; HBD: number of hydrogen bond donors; PSA (Å^2^): polar surface areas; ROTB: number of rotatable bonds; AROM: number of aromatic rings; ALERTS: number of alert structures, which considers a curated reference set of 94 functional moieties that are potentially mutagenic, reactive, or have unfavorable pharmacokinetic properties^43^ property distributions for TTR active/inactive molecules (threshold: 50); (c) Pearson’s correlation coefficients of the correlations between QED properties and TTR binding activities; (d) QED property distributions for Tox24 train/leaderboard/test data sets.

We conducted a comprehensive analysis of the Tox24 challenge data to investigate the distribution of the prediction target (TTR binding activity), chemical structural properties, and their correlations, thereby providing a general understanding of the challenge from a feature analysis perspective. As shown in Figure 2, TTR binding activities in the Tox24 training set and leaderboard test set range from −45 to 111.12%, with a threshold of 50 chosen to classify molecules as active or inactive. According to the Quantitative Estimate of Drug-likeness (QED) properties,^44^ significant differences were observed in the distributions of MW, AlogP, and AROM between active and inactive molecules Figure 2b, indicating that TTR-active molecules tend to have higher molecular weights and greater aromaticity. Correlation analysis results (Figure 2c) revealed weak correlations (Pearson’s correlation coefficients <0.2) between TTR binding activity and MW, AlogP, or AROM, suggesting that simple general properties are not sufficient for accurate prediction. This highlights the need for complex computational modeling to achieve precise TTR binding activity predictions. Furthermore, distribution analysis in Tox24 data sets (train/leaderboard/test) showed similar QED property distributions, indicating consistent chemical structure spaces in all data sets.

Since the physicochemical properties display only weak correlations with TTR toxicity, we further conducted a Bemis–Murcko scaffold-activity analysis to identify representative active scaffolds. As shown in Figure S3, most compounds in the Tox24 training, leaderboard, and test data sets either lack a clearly defined scaffold or consist merely of a simple benzene ring. Moreover, analyses of the most frequently occurring scaffolds among active compounds (Figures S4–S6) indicate that no distinctive scaffold or characteristic—aside from significant aromaticity—can be discerned.

**Figure 3:**
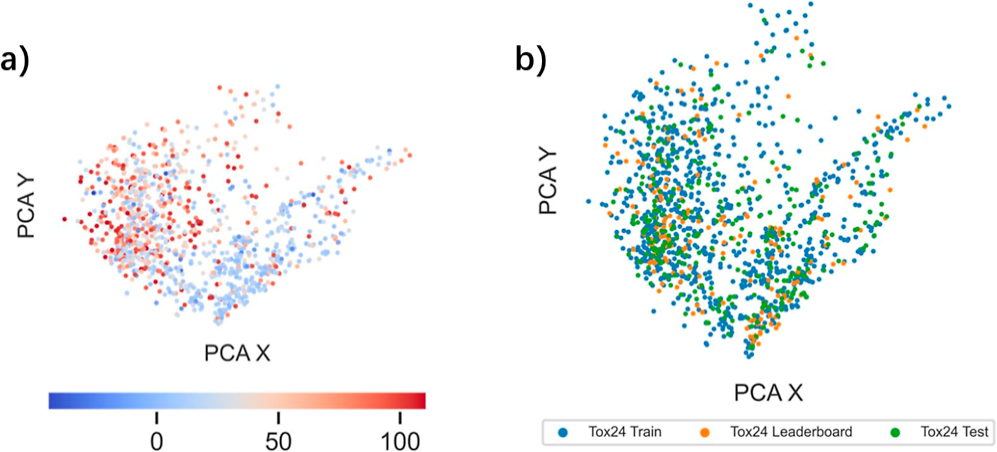
PCA visualizations based on molecular ECFP fingerprints for data in (a) Tox24 leaderboard data sets, with TTR binding activities as scatter colors; (b) Tox24 train/leaderboard/test data sets.

To further explore the chemical structure space of the Tox24 challenge, we calculated 2048 bit ECFP4 fingerprints^45^ for all molecules and applied principal component analysis (PCA) for dimensionality reduction and visualization. As shown in Figure 3a, TTR-active molecules (marked in red) are loosely clustered in the ECFP fingerprint space. Furthermore, the molecules in the Tox24 train, leaderboard, and test data sets are evenly distributed throughout the chemical space, with no clear cluster separation. This finding suggests that the data sets share a similar structural space.

### Performance Comparison on Tox24 Leaderboard and Blind Test Sets

**Figure 4:**
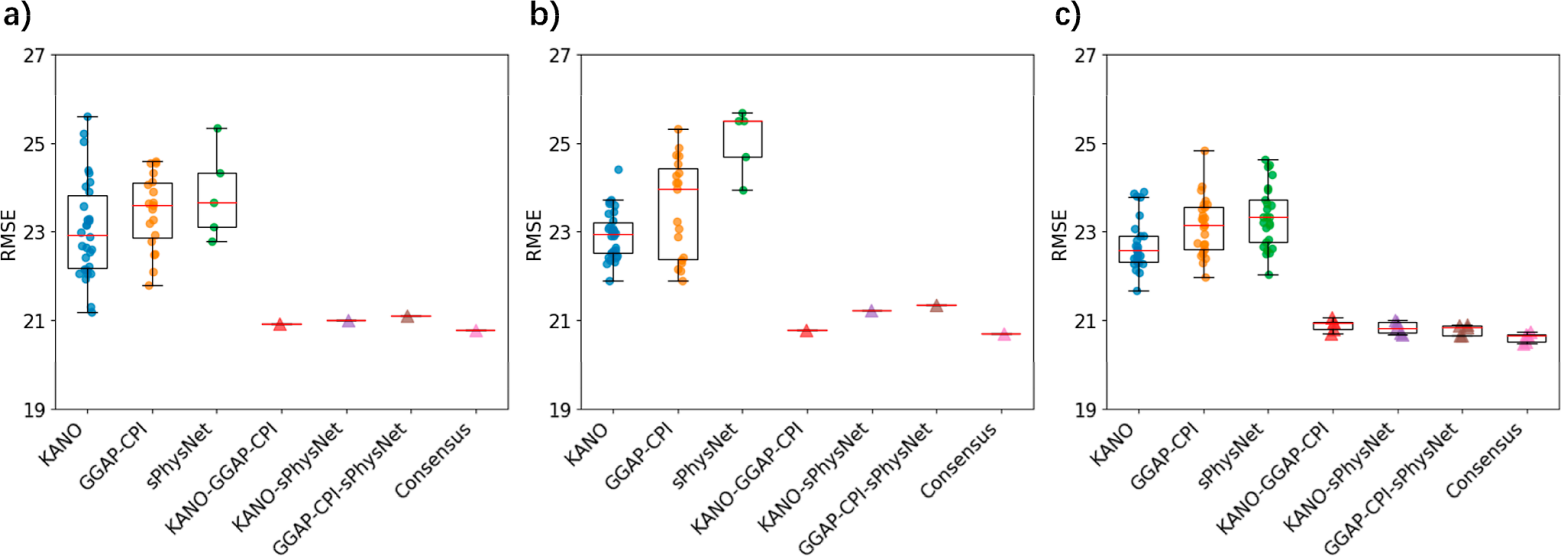
RMSE results for individual models (KANO, GGAP-CPI, and sPhysNet) and consensus models (KANO + GGAP-CPI, KANO + sPhysNet, GGAP-CPI + sPhysNet, Consensus model) on (a) the Tox24 leaderboard test set; (b) the Tox24 blind test set (only trained on training set); (c) the Tox24 test set in a 5 × 5-fold cross-validation offline testing manner that combined the training set and leaderboard test set for model training.

In this challenge, we evaluated our three regression models on the leaderboard test set of 200 compounds and subsequently submitted predictions for the blind test set of 300 compounds. Our novel consensus model achieved a competitive performance, ranking among the top 5 submissions with a root-mean-square error (RMSE) of 20.8 on the blind test set. The performances of each of our individual models (sPhysNet, KANO, and GGAP-CPI) on both the leaderboard and blind test sets are summarized in Figure 4a,b and Tables 1 and 2. Note that the submitted result (RMSE = 20.8) is obtained through soft voting of three ensemble models (the sPhysNet ensemble, KANO ensemble, and GGAP-CPI ensemble). In contrast, the RMSE = 20.7 result in Table 2 is derived from soft voting of all base models directly, without first aggregating individual models before ensembling.

**Table 1:** Model Performance on the Leaderboard Test Set Only Using the Training Set for Model Training

Model	type	*R*-squared	RMSE	MAE	PCC
KANO	ligand-based	0.52 ± 0.05	23.1 ± 1.1	16.8 ± 0.8	0.73 ± 0.02
GGAP-CPI	interaction-based	0.50 ± 0.04	23.5 ± 0.8	16.9 ± 0.7	0.71 ± 0.02
sPhysNet	structure-based	0.48 ± 0.04	23.8 ± 0.9	16.9 ± 0.7	0.71 ± 0.02
KANO + GGAP-CPI	ensemble	0.60	20.9	15.3	0.78
KANO + sPhysNet	ensemble	0.60	21.0	15.1	0.78
GGAP-CPI + sPhysNet	ensemble	0.60	21.1	15.3	0.77
consensus	ensemble	0.61	20.8	15.1	0.78

**Table 2:** Model Performance on the Blind Test Set Only Using the Training Set for Model Training

Model	type	*R*-squared	RMSE	MAE	PCC
KANO	ligand-based	0.59 ± 0.02	22.9 ± 0.5	17.3 ± 0.4	0.77 ± 0.01
GGAP-CPI	interaction-based	0.57 ± 0.04	22.9 ± 0.5	17.3 ± 0.4	0.77 ± 0.01
sPhysNet	structure-based	0.51 ± 0.03	25.1 ± 0.7	18.7 ± 0.5	0.73 ± 0.02
KANO + GGAP-CPI	ensemble	0.66	20.8	15.4	0.82
KANO + sPhysNet	ensemble	0.65	21.2	16.0	0.81
GGAP-CPI + sPhysNet	ensemble	0.64	21.3	15.5	0.81
consensus	ensemble	0.67	20.7	15.3	0.82

To investigate whether combining the models could further improve performance, we evaluated various consensus combinations on both data sets. Across all combinations, consensus models consistently outperformed their single-model counterparts. In particular, combining sPhysNet with either KANO or GGAP-CPI produced superior results compared to the KANO plus GGAP-CPI consensus on both data sets. This finding suggests that the superior performance of sPhysNet plays a key role in the success of these consensus combinations. Furthermore, the full three-model consensus achieved the highest performance compared to the consensus of two models, demonstrating that each model in the consensus contributes complementary information that enhances the overall predictive power for TTR binding affinity. Additionally, we analyzed the correlation of absolute errors among sPhysNet, KANO, and GGAP-CPI on the blind test set. The results show that the Pearson correlation coefficient (PCC) is 0.71 between sPhysNet and KANO, 0.70 between sPhysNet and GGAP-CPI, and 0.81 between KANO and GGAP-CPI (see Figure S1 for details). sPhysNet leverages molecular 3D geometry to model TTR binding information, whereas both KANO and GGAP-CPI rely on molecular topology derived from 2D graphs for their predictions. This difference in the ligand structural modeling and representation may contribute to the variation in predictions among the three models. Figure S2 presents the top 12 molecules with the highest absolute errors for all three models. Notably, mercury(II) chloride (SMILES: [Cl-].[Cl-].[Hg+2], IUPAC name: dichloromercury) exhibits the largest error for KANO and GGAP-CPI. This is likely because the inorganic compound markedly differs from the rest of the training data, and RDKit fails to construct the correct bond connections between the Cl and Hg atoms, preventing the graph neural network from effectively capturing the topology structure for molecular graphs. sPhysNet also struggles with this molecule due to issues with force field parameters and conformation generation.

To maximize the utility of the available data as well as systematically examine the role of consensus learning, we combined the Tox24 training set and leaderboard set as a unified training data set and then conducted a 5 × 5-fold cross-validation offline testing. This integration increased the training data size by approximately 20% compared to our initial submission. The model performance was subsequently evaluated with the Tox24 blind test set. The offline results, presented in Figure 4c and Table 3, reveal consistent performance improvements across all single models and consensus models. Notably, the final consensus model achieved an RMSE of 20.56 on the Tox24 test set. The statistical results (pairwise *t*-test) in Tables S1 and S2 also suggest our consensus model consistently outperforms other two-component ensemble models (KANO-GGAP-CPI, KANO-sPhysNet, GGAP-CPI-sPhysNet) and base models (KANO, GGAP-CPI, sPhysNet) with significant differences on each cross-validation testing. We also included a baseline model trained using XGBoost with 2048 bit ECFP fingerprints; the training details are provided in Text S1. The results in Table 3 indicate that the XGBoost model achieved an RMSE of 26.46, which is significantly higher than that of our consensus model. These results highlight the robustness and scalability of our consensus model, which demonstrates the capacity to achieve even greater performance with larger data sets.

**Table 3:** Model Performance on the Test Set Using the Training Set and Leaderboard Test Set for Model Training in a 5 × 5-Fold Cross Validation Offline Testing Manner

Model	type	*R*-squared	RMSE	MAE	PCC
XGBoost	ligand-based	0.453 ± 0.03	26.5 ± 0.7	19.9 ± 0.5	0.68 ± 0.02
KANO	ligand-based	0.64 ± 0.01	21.4 ± 0.1	16.2 ± 0.1	0.80 ± 0.00
GGAP-CPI	interaction-based	0.64 ± 0.00	21.4 ± 0.1	15.5 ± 0.1	0.80 ± 0.00
sPhysNet	structure-based	0.63 ± 0.01	21.7 ± 0.2	16.2 ± 0.2	0.80 ± 0.00
KANO + GGAP-CPI	ensemble (CV-wise)	0.66 ± 0.00	20.9 ± 0.1	15.4 ± 0.1	0.81 ± 0.00
KANO + sPhysNet	ensemble (CV-wise)	0.66 ± 0.00	20.8 ± 0.1	15.7 ± 0.1	0.82 ± 0.00
GGAP-CPI + sPhysNet	ensemble (CV-wise)	0.66 ± 0.00	20.8 ± 0.1	15.3 ± 0.1	0.82 ± 0.00
consensus	ensemble (CV-wise)	0.67 ± 0.00	20.6 ± 0.1	15.3 ± 0.1	0.82 ± 0.00
KANO + GGAP-CPI	ensemble (all)	0.66	20.8	15.4	0.81
KANO + sPhysNet	ensemble (all)	0.66	20.8	15.6	0.82
GGAP-CPI + sPhysNet	ensemble (all)	0.67	20.7	15.3	0.82
consensus	ensemble (all)	0.67	20.6	15.2	0.82

**Figure 5:**
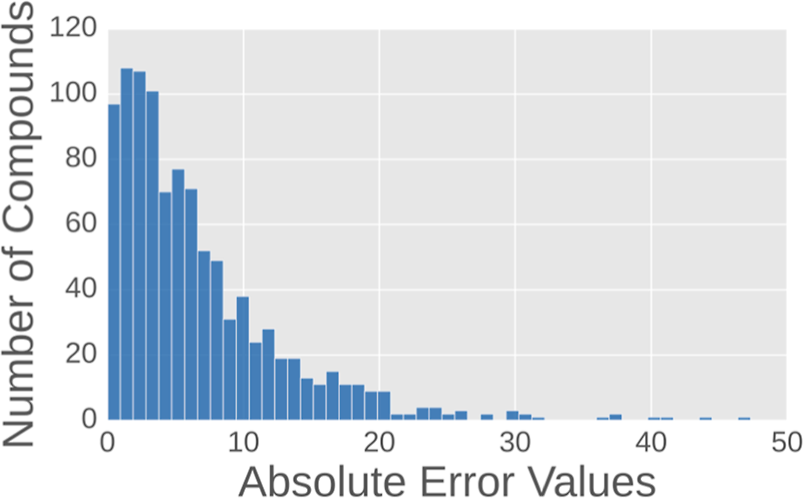
Distribution of absolute errors in the training set.

Although our consensus model performed well on the blind set, the RMSE remained relatively high. We analyzed the absolute error distribution across the training set to explore potential approaches for improving our model performance. We used the model trained only on the training set to calculate the prediction error on the training set. Figure 5 presents the details of the absolute error distribution. On the training set, 21% of the data points have absolute errors greater than 10, 10% exceed 15, and 4% exceed 20; this may represent some noise in the training data. In general, deep learning models tend to achieve very low prediction errors on the training set because of overfitting. We believe that certain factors such as experimental noise or intrinsic compound complexity make the data difficult to learn. Nowadays, denoising learning techniques^46−51^ have been employed in deep learning models to improve performance, using specialized algorithms to identify noisy data and correct or filter it out. We anticipate that applying such methods will further improve our model’s performance in the future.

### Uncertainty Analysis

**Figure 6:**
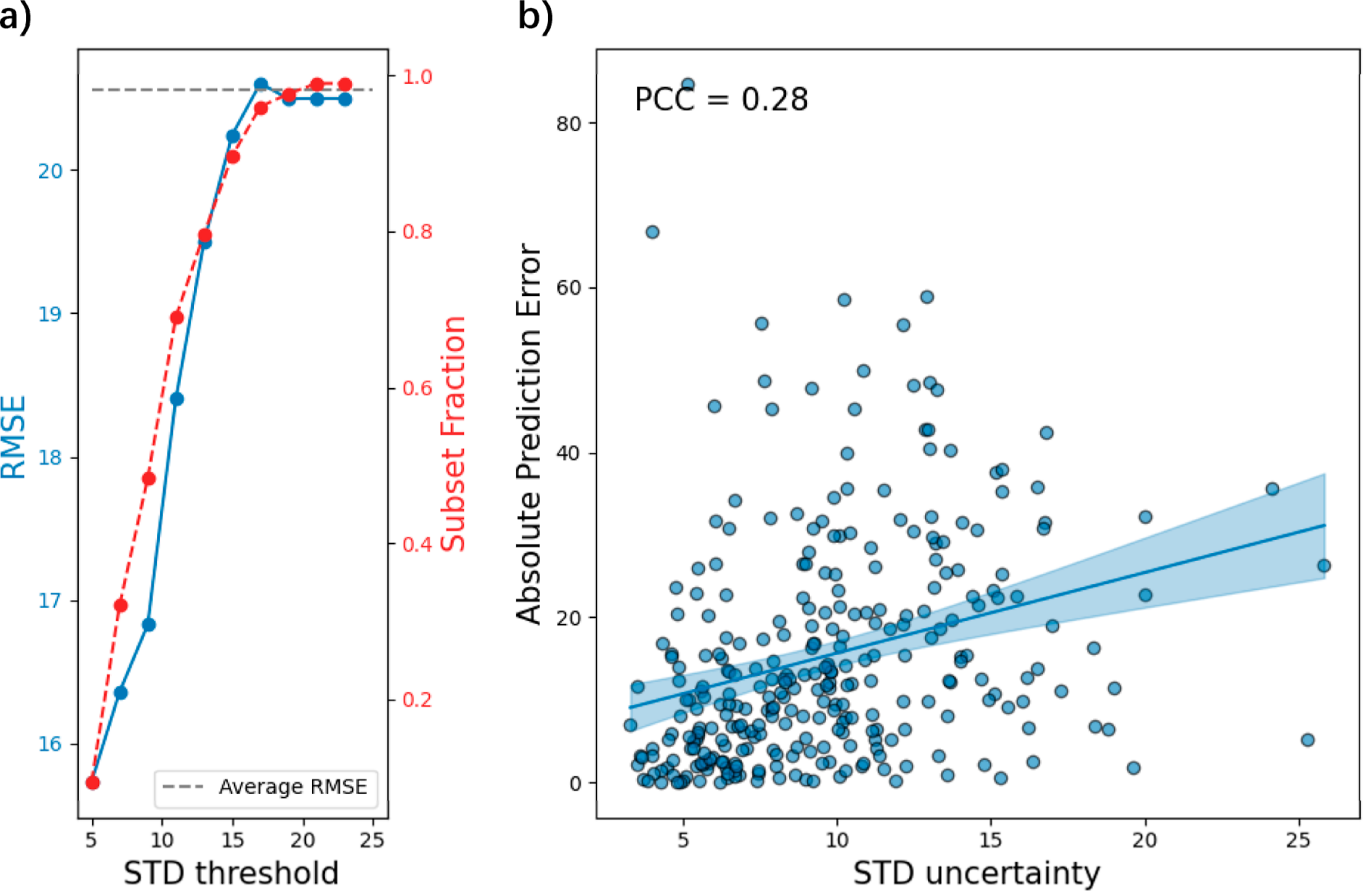
Uncertainty analysis on the Tox24 test set in offline testing. (a) Subset RMSE vs uncertainty threshold. The corresponding uncertainty serves as the threshold to screen samples with lower uncertainty, and then RMSE is calculated on these subset data; (b) individual absolute prediction error vs uncertainty values.

Uncertainty estimation has been an increasingly popular research topic with the advancement of artificial intelligence as researchers often seek to gauge the confidence of their predictions in a systematic manner.^52−55^ In this work, our consensus model serves not only to improve the performance of our submission but also to provide an estimate of prediction uncertainty. The standard deviation captures the variability across the multiple predictions; it also provides an effective estimate of predictive uncertainty for an ensemble model.^56^ The mean value of all predictions is employed as the final result, and the standard deviation is utilized as the uncertainty measure for our consensus model. Figure 6a illustrates the relationship between the uncertainty and the RMSE on the test set. Statistically, the prediction RMSE increases as model uncertainty rises. Notably, over 80% of compounds in the test set exhibit an average RMSE below 20 when using an uncertainty threshold of 15. This suggests that compounds with higher estimated uncertainty tend to have larger prediction errors compared to those with lower uncertainty. However, the compound-level relationship between prediction error and uncertainty shows a weak correlation (PCC = 0.28), which may be influenced by rare structures, such as the molecule [Cl^–^].[Cl^–^].[Hg^2+^] that has an error of 84.73. These findings indicate that the uncertainty derived from our consensus model can potentially serve as a reference for assessing the quality of our predictions. Determining the applicability domains of a model is crucial, and various distance-to-model metrics can be employed for this purpose.^57,58^ Tetko et al. conducted a systematic study of the effectiveness of using the standard deviation of model predictions to characterize the distance from an ensemble model. Their findings indicate that standard deviation is an efficient metric for quantifying the distance to the models.^59^ We believe that the standard deviation derived from our consensus model can similarly serve as a valuable metric for evaluating its applicability to domains.

## Conclusions

TTR is an important component of TH transport and homeostasis in blood and target tissues. In this challenge, leveraging the biggest data set provided by the organizers, we developed a consensus model that integrates sPhysNet, KANO, and GGAP-CPI to predict TTR binding affinity. Each model utilized a different level of molecular information including 2D topology, 3D geometry, and protein–ligand interactions. This consensus model performed well on the blind test set, yielding an RMSE of 20.8 and ranking in the top 5. After the leaderboard set was incorporated into our training data, the RMSE improved to 20.6. The results demonstrate that combining three regression models enhances the predictive accuracy. Furthermore, by analyzing the model uncertainty, we observed that both the RMSE and the prediction interval error increased as uncertainty rose. Thus, the uncertainty measure provided by our consensus model might be a useful reference for estimating the confidence associated with each prediction. An analysis of the error distribution in the training set revealed that many molecules exhibit large prediction errors, suggesting that the deep learning model does not fit them very well for the training data, potentially due to experimental noise. We believe that model performance can be further improved by employing a denoising learning method, incorporating additional data, and integrating more structure-based information as prior knowledge. We are confident that this consensus model can serve as a valuable resource for identifying potential TTR binders and predicting their binding affinities in silico.

## Data Availability

All data sets and source codes in this work are available. The data set from Tox24 challenge can be accessed at https://ochem.eu/static/challenge.do. All source codes for data set preparation, model training, and model usage are at https://github.com/xiaolinpan/tox24_challenge_submission_yingkai_lab.

## Supporting Information Available

Description of training for the baseline models; correlation of absolute errors among sPhysNet, KANO, and GGAP-CPI on the blind test set; visualization of 12 molecules with the highest absolute errors on the blind test set for three models; top 10 scaffolds with counts of active and inactive compounds in the Tox24 training set, the Tox24 leaderboard set, and the Tox24 test set; top 10 scaffolds in the Tox24 training set; top 10 scaffolds in the Tox24 leaderboard set; top 10 scaffolds in the Tox24 test set; statistical significance of pairwise RMSE performances on the test set using the training set and leaderboard test set for model training; and number of cross-validation folds (out of 5) in which the model listed in the “Model” column achieved a lower RMSE compared to the other models (PDF)
