# Supplementary material for: Enhancing Transthyretin Binding Affinity Prediction with a Consensus Model: Insights from the Tox24 Challenge
Source: Chem Res Toxicol. 2025 Apr 26;38(5):900–8. doi: 10.1021/acs.chemrestox.4c00560 (PMC12093365; doi:10.1021/acs.chemrestox.4c00560)
Supplement: Supplementary file 1 — tx4c00560_si_001.pdf [file tx4c00560_si_001.pdf]

Supporting Information: Enhancing Transthyretin Binding Affinity Prediction with a Consensus Model: Insights from the Tox24 Challenge

Xiaolin Pan[1,*], Yaowen Gu[1], Weijun Zhou[1], Yingkai Zhang[1,2,3,*]

[1]Department of Chemistry, New York University, New York, 10003, United States
[2]Simons Center for Computational Physical Chemistry at New York University, New York, 10003, United States
[3]NYU-ECNU Center for Computational Chemistry at NYU Shanghai, Shanghai, 200062, China

Email: xp2042@nyu.edu, yingkai.zhang@nyu.edu

Table of Contents

Text S1. The description of training details for the baseline model **S3**

Figure S1. The correlation of absolute errors among sPhysNet, KANO, and GGAP-CPI on the blind test set **S3**

Figure S2. Visualization of the 12 molecules with the highest absolute errors on the blind test set for KANO, GGAP-CPI, and sPhysNet, with the displayed values representing the absolute errors...**S4**

Figure S3. Top 10 scaffolds with counts of active and inactive compounds in (a) the Tox24 training set, (b) the Tox24 leaderboard set, and (c) the Tox24 test set................................**S5**

Figure S4. Top 10 scaffolds in the Tox24 training set, ranked by the number of compounds with active TTR toxicity (toxicity > 50). AF denotes the fraction of active compounds in each scaffold, and Avg Tox represents the average TTR toxicity for compounds within that scaffold **S5**

Figure S5. Top 10 scaffolds in the Tox24 leaderboard set, ranked by the number of compounds with active TTR toxicity (toxicity > 50). AF denotes the fraction of active compounds in each scaffold, and Avg Tox represents the average TTR toxicity for compounds within that scaffold..**S6**

Figure S6. Top 10 scaffolds in the Tox24 test set ranked by the number of compounds with active TTR toxicity (toxicity > 50). AF denotes the fraction of active compounds in each scaffold, and Avg Tox represents the average TTR toxicity for compounds within that scaffold **S6**

Table S1. Statistical significance of pairwise RMSE performances on the test set using training set and leaderboard test set for model training...**S7**

Table S2. Number of cross-validation folds (out of 5) in which the model listed in the "Model" column achieved a lower RMSE compared to the other models...........................**S7**

Text S1. The description of training details for the baseline model.

To fairly compare the superiority of our consensus model with baseline (XGBoost), as clarify previous inconsistent training setting of each individual model, we have now conduct a 5-fold cross validation study on all of our base models, as well as a XGBoost with well-tuned hyperparameters (search space: 'max_depth': [3, 4, 5, 6, 7], 'learning_rate': [0.01, 0.05, 0.1, 0.2, 0.3], 'n_estimators': [100, 200, 300, 400, 500]). The results suggest that XGBoost with ECFP2048 fingerprints could achieve a RMSE of 26.46±0.66, which is significantly lower than our consensus model. A jupyter notebook for reproducing the baseline results can be found at: https://github.com/xiaolinpan/tox24_challenge_submission_yingkai_lab/blob/main/tox24_XGB.ipynb

Figure S1. The correlation of absolute errors among sPhysNet, KANO, and GGAP-CPI on the blind test set.

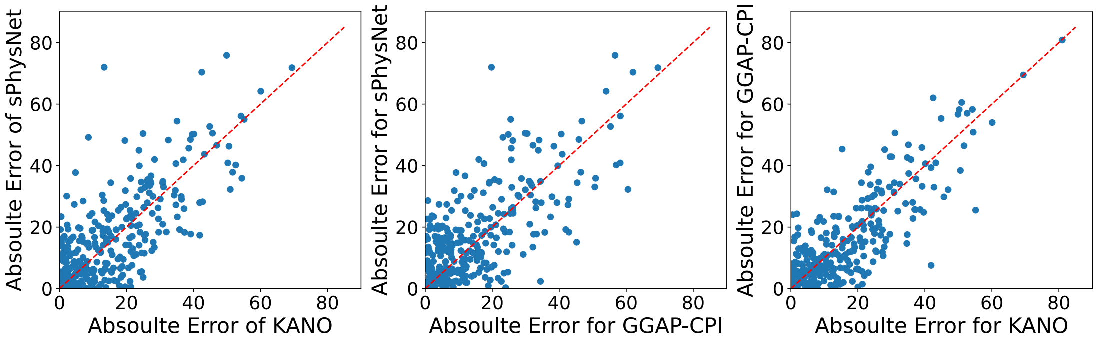

Figure S2. Visualization of the 12 molecules with the highest absolute errors on the blind test set for KANO, GGAP-CPI, and sPhysNet, with the displayed values representing the absolute errors.

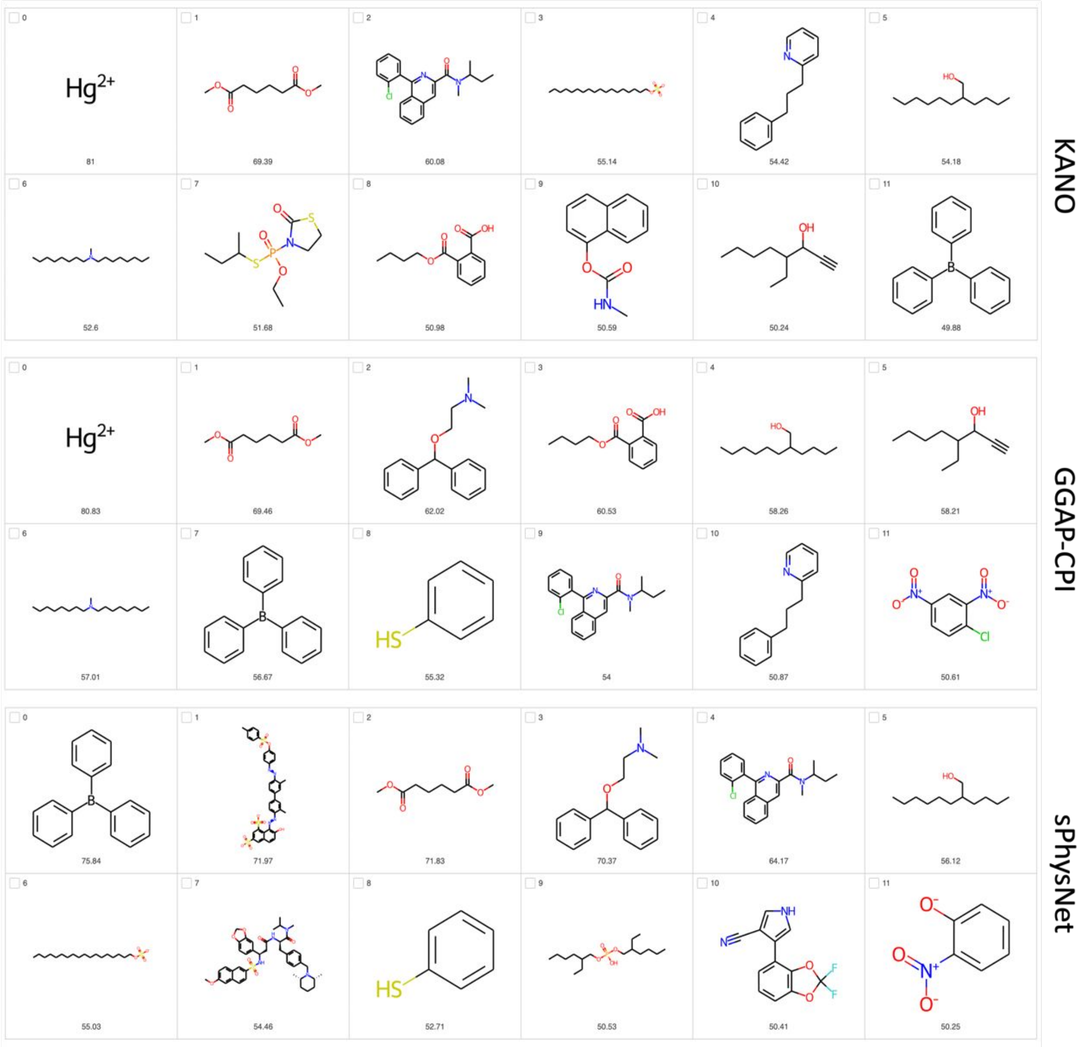

Figure S3. Top 10 scaffolds with counts of active and inactive compounds in (a) the Tox24 training set, (b) the Tox24 leaderboard set, and (c) the Tox24 test set.

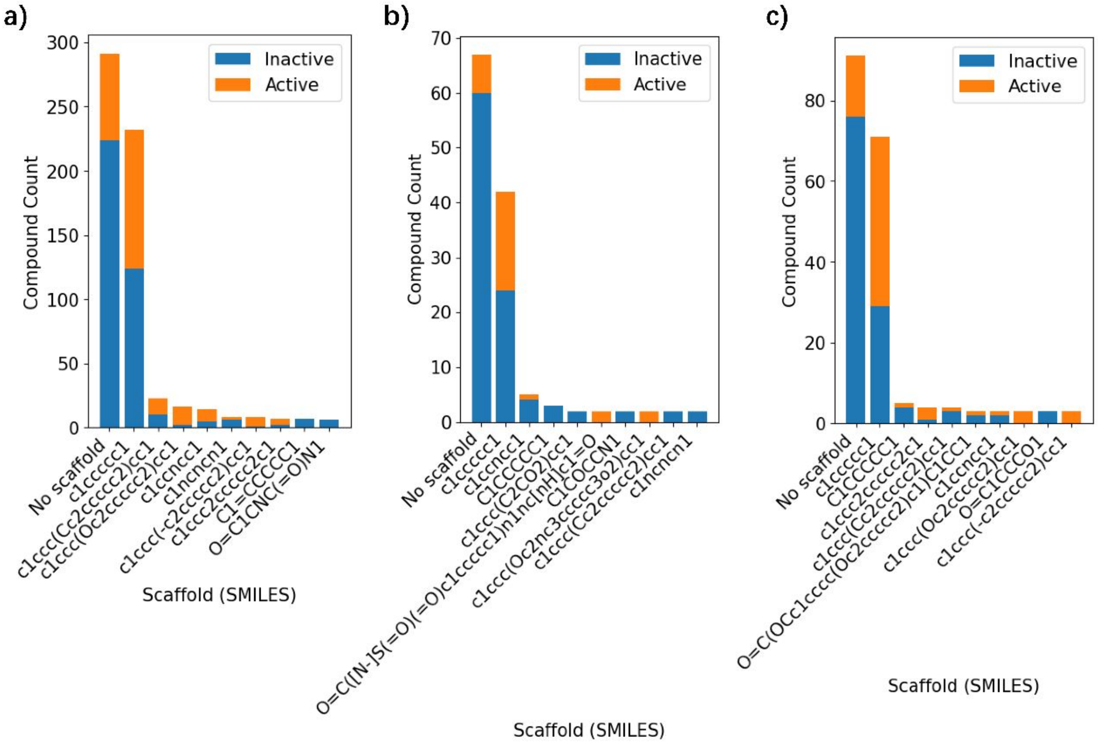

Figure S4. Top 10 scaffolds in the Tox24 training set, ranked by the number of compounds with active TTR toxicity (toxicity > 50). AF denotes the fraction of active compounds in each scaffold, and Avg Tox represents the average TTR toxicity for compounds within that scaffold.

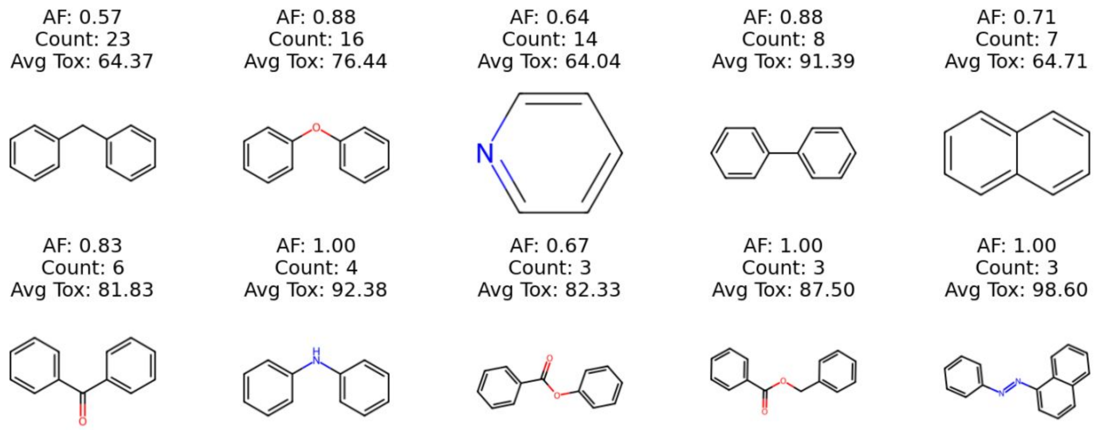

Figure S5. Top 10 scaffolds in the Tox24 leaderboard set, ranked by the number of compounds with active TTR toxicity (toxicity > 50). AF denotes the fraction of active compounds in each scaffold, and Avg Tox represents the average TTR toxicity for compounds within that scaffold.

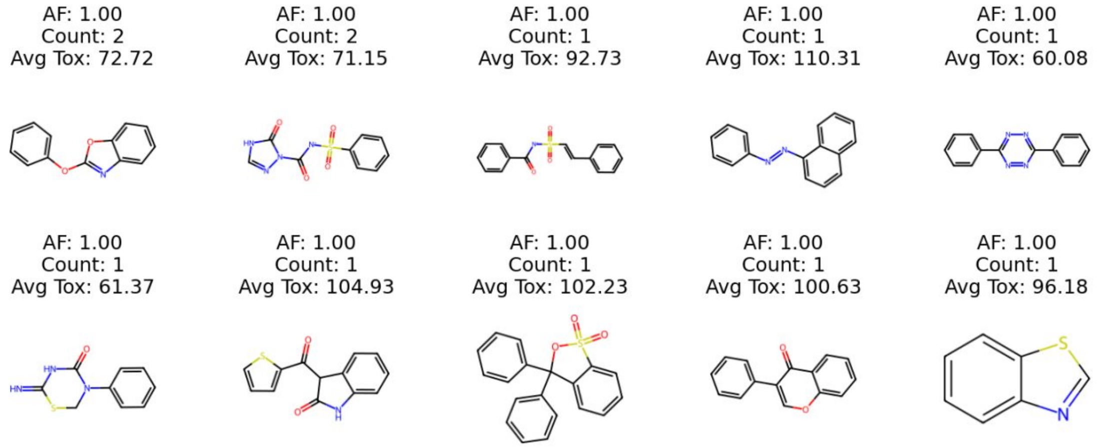

Figure S6. Top 10 scaffolds in the Tox24 test set ranked by the number of compounds with active TTR toxicity (toxicity > 50). AF denotes the fraction of active compounds in each scaffold, and Avg Tox represents the average TTR toxicity for compounds within that scaffold.

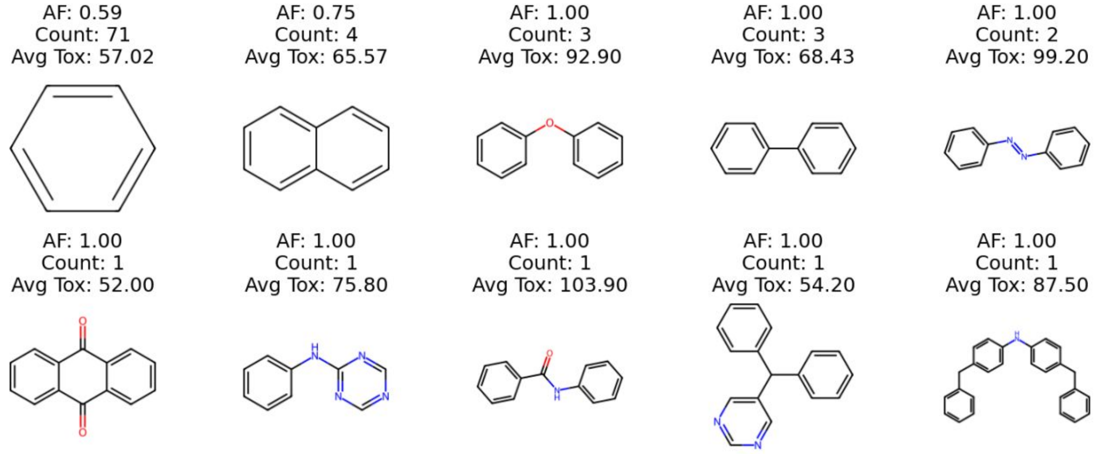

Table S1. Statistical significance of pairwise RMSE performances on the test set using training set and leaderboard test set for model training.

Model	KANO	GGAP-CPI	sPhysNet	KANO+ GGAP-CPI	KANO+ sPhysNet	GGAP-CPI+ sPhysNet
KANO	-	-	-	-	-	-
GGAP-CPI	0.74	-	-	-	-	-
sPhysNet	0.05	0.05	-	-	-	-
KANO+GGAP-CPI	<0.001	<0.001	0.002	-	-	-
KANO+sPhysNet	0.001	0.002	<0.001	0.49	-	-
GGAP-CPI+sPhysNet	0.002	<0.001	<0.001	0.18	0.28	-
Ensemble	<0.001	<0.001	<0.001	0.004	0.003	0.002

Table S2. Number of cross-validation folds (out of 5) in which the model listed in the "Model" column achieved a lower RMSE compared to the other models.

Model	KANO	GGAP-CPI	sPhysNet	KANO+ GGAP-CPI	KANO+ sPhysNet	GGAP-CPI+ sPhysNet
KANO	-	2.0	4.0	0.0	0.0	0.0
GGAP-CPI	3.0	-	5.0	0.0	0.0	0.0
sPhysNet	1.0	0.0	-	0.0	0.0	0.0
KANO+GGAP-CPI	5.0	5.0	5.0	-	2.0	1.0
KANO+sPhysNet	5.0	5.0	5.0	3.0	-	1.0
GGAP-CPI+sPhysNet	5.0	5.0	5.0	4.0	4.0	-
Ensemble	5.0	5.0	5.0	5.0	5.0	5.0
